# Study on Nano-Grinding Characteristics and Formation Mechanism of Subsurface Damage in Monocrystalline Silicon

**DOI:** 10.3390/mi16090976

**Published:** 2025-08-25

**Authors:** Haipeng Yan, Haining Zhang, Siyuan Cao, Chao Wang

**Affiliations:** 1 School of Mechanical Engineering, Hebei University of Science and Technology, Shijiazhuang 050018, China; lnyanhp@126.com; 2 Center for Innovation and Practice, Baotou Iron & Steel Vocational Technical College, Baotou 014010, China; haiyanghaiyao@163.com; 3 School of Mechanical Engineering, Shenyang Institute of Engineering, Shenyang 110136, China

**Keywords:** nano-grinding, molecular dynamics, monocrystalline silicon, grinding temperature, subsurface damage

## Abstract

Monocrystalline silicon is an excellent semiconductor material for integrated circuits. Its surface quality has an enormous effect on its service life. The surfaces are formed by ultra-precision machining using nano-grinding, one of the technologies that can achieve surface roughness at the nano- or sub-nano-scale. Therefore, subsurface damage of monocrystalline silicon in nano-grinding was studied by establishing a molecular dynamics simulation model, and the impact of machining parameters on the force–thermal behavior was analyzed. The results reveal that the mechanism of subsurface damage is mainly structural phase transformation and amorphization. In nano-grinding of monocrystalline silicon, the tangential grinding force has a relatively major role in material removal. With increasing grinding depth and grinding speed, the grinding heat rises, and a certain degree of high temperature strengthens the toughness of the material, improving the subsurface quality of monocrystalline silicon. Therefore, subsurface damage in monocrystalline silicon can be controlled by reducing the grinding depth and increasing the grinding speed.

## 1. Introduction

Monocrystalline silicon, as a semiconductor material, is widely used in the manufacturing of integrated circuits due to its outstanding properties, such as high-temperature resistance, oxidation resistance, wear resistance, and high strength. It is also considered a basic component of many electronic devices, including mobile phones, computers, and appliances [1]. In integrated-circuit manufacturing technology, the requirements for high performance, multifunctionality, miniaturization, and low power consumption of products are becoming increasingly stringent, which places greater demands on the planarization and thinning technology of silicon wafers [2]. At present, grinding is commonly utilized in the thinning and planarization of silicon wafers because of its ability to improve the material removal rate at a low cost [3]. Due to the machining precision at the micron level in ordinary grinding, subsurface damage such as crystal defects and micro-cracks inevitably occurs in the workpiece, thereby restricting further processing of silicon wafers [4]. However, nano-grinding technology has been widely employed for ultra-precision machining of monocrystalline silicon surfaces due to its ability to achieve nano- or sub-nano-scale surface roughness and near-atomic-scale subsurface damage [5,6].

The force–thermal behavior has an important influence on subsurface damage in silicon wafers during nano-grinding. Due to the machining precision at the nano level, ordinary methods cannot effectively detect the grinding force and grinding heat, or even represent the interaction mechanism between an abrasive grain and monocrystalline silicon. Molecular dynamics (MD) simulation has been gradually applied to the analysis of material processing at the nano-scale or atomic level, because it can monitor the force–thermal behavior and deformation process of the materials during workpiece processing in situ in real time [7]. Many researchers have studied the subsurface formation mechanism during nano-grinding of semiconductor materials, such as monocrystalline silicon, using an MD model. Li et al. [1] employed MD model simulations to investigate the damage mechanisms in monocrystalline silicon under varying grinding speeds. Their results demonstrated an inverse correlation between grinding speed and grinding force. Furthermore, Li et al. [8] established an MD model for single-crystal scratching, analyzing dislocation nucleation and slip mechanisms during monocrystalline silicon grinding. This work provided a theoretical foundation for predicting and controlling dislocation damage in ultra-precision grinding of monocrystalline silicon. Subsequently, Liu et al. [9] integrated MD simulations with rotational wafer grinding processes, successfully mapping the residual stress distribution in silicon wafers. Concurrently, Li et al. [10] combined slope nano-scratch experiments with MD simulations to systematically study deformation behavior and dislocation evolution in monocrystalline silicon, providing theoretical guidance for defect control in ultra-precision machining. Yan et al. [11] examined the effects of cutting speed and tool geometry on subsurface damage in monocrystalline silicon through MD modeling, revealing that subsurface damage formation is primarily driven by phase transformation. Their findings indicate that excessive negative tool rake angles and reduced grinding speeds both contribute to increased subsurface damage depth. Zhang et al. [12] employed MD simulations to examine nano-grinding-induced damage in gallium nitride across varying initial temperatures. Their results revealed that elevated initial temperatures reduce grinding forces but simultaneously increase dislocation density, the quantity of amorphous atoms, and ultimate tensile strength. Meanwhile, Liu et al. [13] elucidated the nano-scale subsurface crystal damage formation mechanism during elliptical vibration cutting of monocrystalline silicon through MD modeling, demonstrating that under high-temperature conditions, a marked rise in Shockley partial dislocation density is detected. In a separate study, Zhao et al. [14] investigated subsurface damage using different abrasive grain geometries via MD simulations, finding that tetrahedral abrasive grains minimize subsurface dislocation formation in nano-grinding. Parallel work by Zhao et al. [15] explored crystal orientation effects on subsurface damage in crystalline materials through MD modeling, revealing significant variations in damage severity across different crystallographic orientations and showing improved processing quality of monocrystalline silicon with {110} surface crystal orientation.

The above studies have enriched the understanding of nano-grinding of semiconductor materials such as monocrystalline silicon and gallium nitride, but most studies focus on discussing the deformation mechanism during material removal, subsurface damage, and the influence of machining parameters on subsurface damage. The force–thermal behavior and potential energy change are not analyzed in these studies. There is a close relationship among grinding force, grinding temperature, friction coefficient, shear stress, subsurface damage, and potential energy in the process of monocrystalline silicon nano-grinding [16]. The magnitude of grinding force directly affects the change in grinding temperature [17]. A large grinding force usually generates more heat, resulting in an increase in grinding temperature [18]. The change in grinding temperature also affects the friction coefficient and changes the friction characteristics of the material during grinding [19]. In addition, the shear stress is closely related to the grinding force and is an important factor affecting material removal [20]. The change in potential energy reflects the interaction between atoms, which may lead to the occurrence of subsurface damage [21]. Therefore, an MD simulation model was developed to explore the effect of force–thermal behavior on the subsurface damage and potential energy situation in monocrystalline silicon nano-grinding, providing a theoretical basis for the suppression of subsurface damage in monocrystalline silicon.

## 2. Materials and Methods

**Figure 1:**
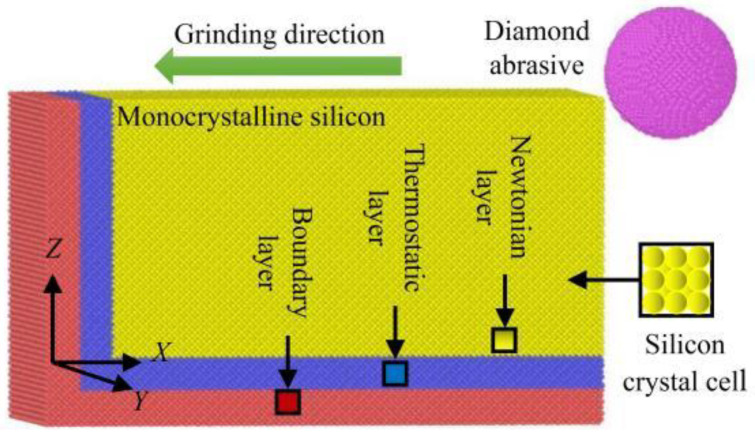
An MD model for nano-grinding in monocrystalline silicon.

A method based on MD simulation was used to explore the characteristics of nano grinding in monocrystalline silicon. The simulation model is shown in Figure 1. The workpiece is monocrystalline silicon with a cubic diamond structure, mainly composed of a Newtonian layer, a thermostatic layer, and a boundary layer [22]. The Newtonian layer makes atoms obey Newton’s second law, and integrates them through the velocity Verlet algorithm to better reflect the interaction between atoms and abrasive grains. The thermostatic layer realizes the temperature control of the model and allows for heat exchange between the workpiece and the outside. To avoid the situation of high-temperature instability in the actual processing, its internal atoms also conform to Newton’s second law [23]. The boundary layer holds the whole model in place and prevents slippage due to force in the grinding [24]. The abrasive grains are simplified diamond abrasives, which are set as spherical and rigid bodies. For boundary conditions in the model, both the *x* and *z* directions are non-periodic, and the *y* direction is periodic [25]. In addition, the *x*-axis direction indicates the [100] crystal direction, the *y*-axis direction indicates the [010] crystal direction, and the *z*-axis direction indicates the [001] crystal direction in crystallography.

Before simulating the nano-grinding, the initial configuration is often not the lowest energy state, and there is unreasonable contact and excessive energy between atoms. Based on the relaxation process in the NVE ensemble, the system can naturally adjust its internal structure through the interaction between atoms without external energy exchange, so that the energy of the system gradually decreases and the system becomes stable. Therefore, a 60 ps relaxation of the model system under the NVE was performed to achieve energy minimization. The initial temperature was adjusted to 300 K by the thermostatic layer [5]. The model was simulated under the NVT system synthesis considering the requirements of volume control, computational efficiency, and stability. In the model, there are two kinds of atoms, C and Si, and three types of inter-atomic interactions (Si-Si, Si-C, and C-C). The interaction between C and C is negligible because the diamond abrasives were set as rigid bodies. The Tersoff function is used for both Si-C and Si-Si interactions [26,27], and the relevant equations are expressed in Equations (1)–(5). The MD simulations were carried out in the software LAMMPS (2Aug2023 version) [28]. However, it lacks visualization capabilities. So, the OVITO (Version 3.9.0) open-source software was adopted for the visualization and analysis of the latter data [29]. The specific parameters for simulation are listed in Table 1.

(1) $$E=\frac{1}{2}\sum_{i\neq j}V_{ij},$$

(2) $$V_{ij}=f_{c}(r_{ij})[f_{R}(r_{ij})+b_{ij}f_{A}(r_{ij})]$$

(3) $$f_{R}(r_{ij})=A_{ij}\exp(-\lambda_{ij}r_{ij}),$$

(4) $$f_{A}(r_{ij})=-B_{ij}\exp(-\mu_{ij}r_{ij}),$$

(5) $$f_{c}(r_{ij})=\left\{\begin{array}{cc} 1 & r_{ij}\leq R_{ij}\\ \frac{1}{2}+\frac{1}{2}\cos[\pi \left(r_{ij}-R_{ij}\right)/\left(S_{ij}-R_{ij}\right)] & R_{ij}<r_{ij}\leq S_{ij},\\ 0 & r_{ij}>S_{ij} \end{array}\right.$$

where *E* indicates the total potential energy for the system; *V_ij_* indicates the interaction energy between atom *i* and *j*; *A_ij_*, *B_ij_*, *λ_ij_*, *μ_ij_* are the characteristic parameters in the potential function; *f_R_* is the repulsive energy; *f_A_* is the attractive energy; *r_ij_* indicates the distance between atom *i* and *j*; *f_c_* represents the cutoff function; and *R_ij_*, *S_ij_* are the cutoff radii.

The modulation function *b_ij_* is expressed as:

(6) $$b_{ij}=\chi_{ij}{\left(1+\beta_{i}^{n_{i}}\zeta_{ij}^{n_{i}}\right)}^{-1/2n_{i}},$$

(7) $$\zeta_{ij}=\sum_{k\neq i,j}f_{c}(r_{ik})\omega_{ik}g(\theta_{ijk}),$$

(8) $$g(\theta_{ijk})=1+\frac{c_{i}^{2}}{d_{i}^{2}}-\frac{c_{i}^{2}}{d_{i}^{2}+(h_{i}-\cos\theta_{ijk})},$$

where *ζ_ij_* represents the angular potential energy; *θ_ijk_* represents the bond angles between atoms; *d_i_*, *c_i_*, and *h_i_* are the correction coefficients related to the potential function; and *β_i_* represents the key level coefficient. The parameter settings in the above equations can be found in Ref. [27].

For the study, the reverse heat transfer sequential algorithm was used to calculate the heat flux density. The estimated formula for heat flux density *q_K_* at step *K* [30] is

(9) $$q_{K}=\sum_{j=0}^{r-1}\left(T_{K+j}-\sum_{n=0}^{K-1}q_{n}\Delta \varphi_{K-n+j}-T_{0}\right)\times \frac{\sum_{l=0}^{j}\Delta \varphi_{j-1}}{\sum_{j=0}^{r-1}{\left(\sum_{l=0}^{j}\Delta \varphi_{j-1}\right)}^{2}}$$

where *q_K_* is heat flux; *T_K_* + *j* is the temperature at *K* + *j*; *q_n_* is the heat flux in the previous step; *T*_0_ is the initial temperature; ∆*φ* indicates the temperature change. The temperature is calculated based on the average kinetic energy equation (see Ref. [25]).

## 3. Results and Discussion

### 3.1. Analysis of Nano-Grinding Properties in Monocrystalline Silicon

#### 3.1.1. Change in Force–Thermal During Nano-Grinding

**Figure 2:**
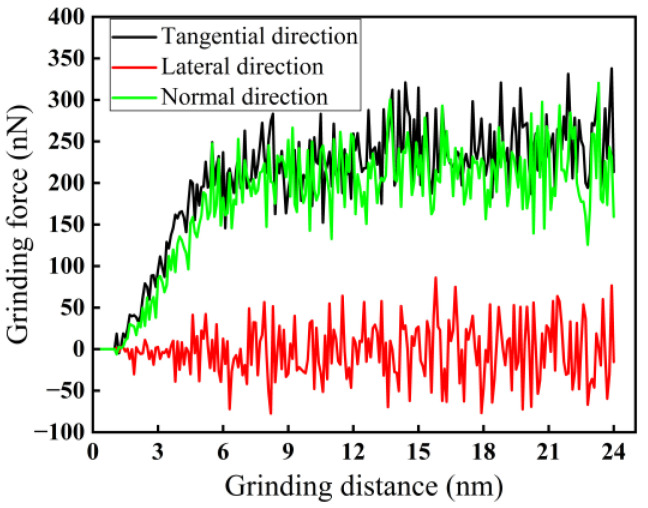
Variation curves of force in nano-grinding of monocrystalline silicon (*d* = 2.0 nm, *v* = 200 m/s).

For a grinding speed of 200 m/s and a grinding depth of 2.0 nm, the variation curves of grinding force in three directions are shown in Figure 2. As demonstrated in Figure 2, both the forces in tangential and normal directions applied by the abrasive grains first increase rapidly during the grinding process, and then fluctuate steadily, while the lateral grinding force has no obvious change and always fluctuates near zero. It can also be observed that the force in the tangential direction is higher than that in the normal direction. Even if the two grinding forces have a minimum difference value, it still reflects that the tangential grinding force has a crucial role in material removal during the nano-grinding process of monocrystalline silicon [31]. In addition, in nano-grinding, the local plastic deformation of materials will lead to changes in grinding force. Because the lateral grinding force fluctuates only near zero, indicating that it has no effect on material removal, it is ignored in the subsequent analysis. When the grinding distance is between 6 and 24 nm, there is a stable grinding zone for a grinding speed of 200 m/s and a grinding depth of 2.0 nm.

**Figure 3:**
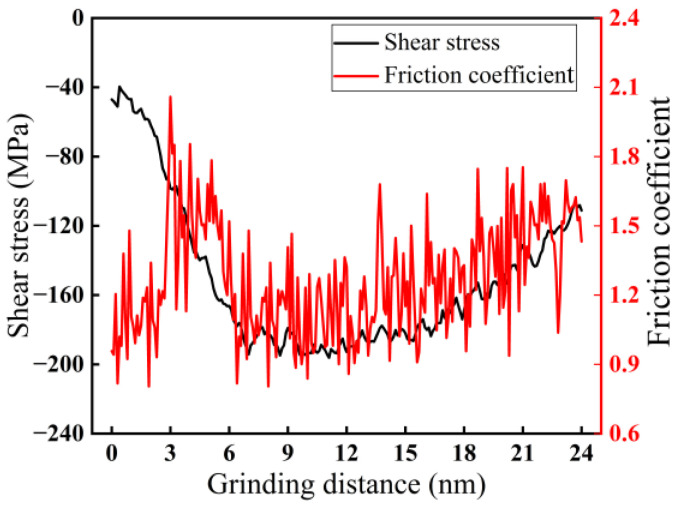
Variation curves of shear stress and friction coefficient with grinding distance (*d* = 2.0 nm, *v* = 200 m/s).

In the grinding process, the abrasive grains apply force to the surface of monocrystalline silicon to remove the material. Through the action of grinding force, two key parameters, shear stress and friction coefficient, are generated. The friction coefficient has a significant influence on the formation of the subsurface and the stability in nano-grinding [32], and shear stress is a key factor in the plastic deformation of monocrystalline silicon [33]. The variation curves of shear stress and friction coefficient with grinding distance in nano-grinding of monocrystalline silicon are presented in Figure 3. As shown in Figure 3, the shear stress is negative (compressive stress), and with increasing grinding distance, the shear stress first increases and then decreases. The reason may be that the distribution and transmission of stress have a lag. The friction coefficient fluctuates within a certain range, indicating that the grinding process is unstable. The reason may be that the plastic deformation of monocrystalline silicon in nano-grinding leads to subsurface damage, resulting in fluctuations in the friction coefficient.

**Figure 4:**
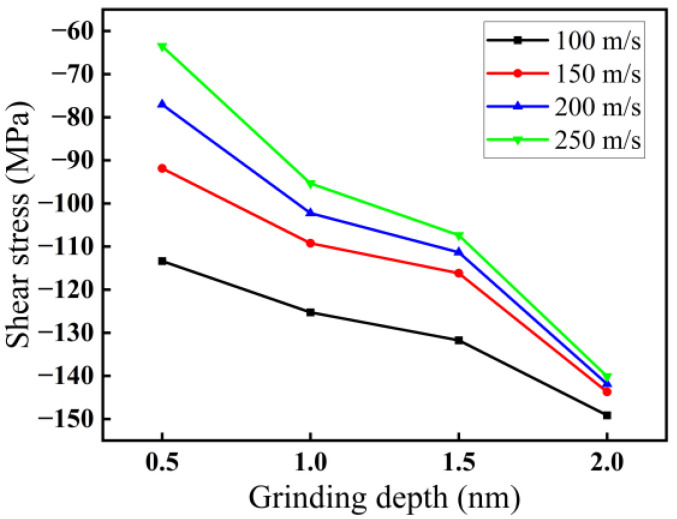
Variations in shear stress for four speeds: 100, 150, 200, and 250 m/s with grinding depth.

The variations in shear stress with grinding depth and grinding speed are demonstrated in Figure 4. The values in Figure 4 are the average values in the stable grinding stage. As demonstrated in Figure 4, the absolute value of the shear stress increases with increasing grinding depth and decreasing grinding speed. For a grinding speed of 200 m/s, when the grinding depth increases from 0.5 to 2.0 nm, the absolute value of the shear stress increases from 77 to 142 MPa. For a grinding depth of 2.0 nm, when the grinding speed increases from 100 to 250 m/s, the absolute value of the shear stress decreases from 149 to 140 MPa. The reason may be that increasing the grinding speed reduces the grinding force, so with increasing grinding speed, the absolute value of the shear stress also decreases. However, the grinding force becomes larger with increasing grinding depth, so the absolute value of the shear stress also increases.

**Figure 5:**
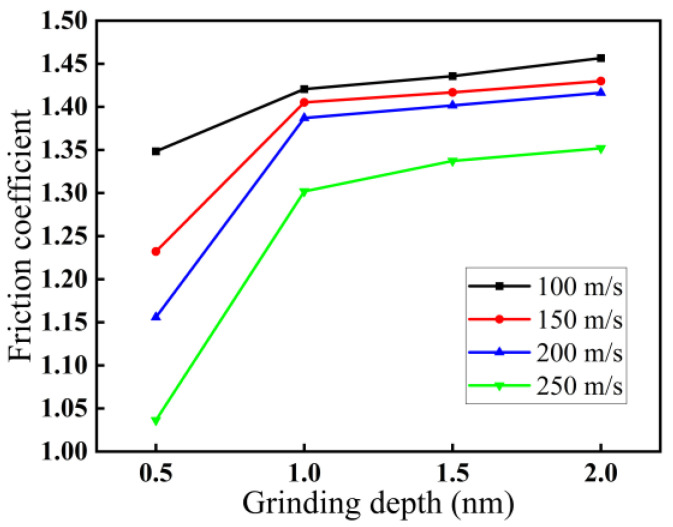
Variations in friction coefficient for four speeds: 100, 150, 200, and 250 m/s with grinding speed.

The variations in the friction coefficient with grinding depth and grinding speed are demonstrated in Figure 5. The values in Figure 5 are also the average values of the stable grinding stage. As demonstrated in Figure 5, the friction coefficient increases with increasing grinding depth and decreasing grinding speed. For a grinding speed of 200 m/s, when the grinding depth increases from 0.5 to 2.0 nm, the friction coefficient increases from 1.16 to 1.42, which indicates that the larger the grinding depth, the worse the stability and the greater the subsurface damage. For a grinding depth of 2.0 nm, when the grinding speed increases from 100 to 250 m/s, the friction coefficient decreases from 1.46 to 1.35, indicating that the higher the grinding speed, the better the stability and the less subsurface damage. The reason may be that as the grinding depth increases, the amount of material removed per unit time increases, resulting in an increase in the roughness of the workpiece surface and grinding resistance. Therefore, the friction coefficient also increases accordingly. However, the heat increases with increasing grinding speed, causing the temperature of the workpiece surface to rise and the material surface hardness to soften, resulting in a corresponding decrease in the friction coefficient.

**Figure 6:**
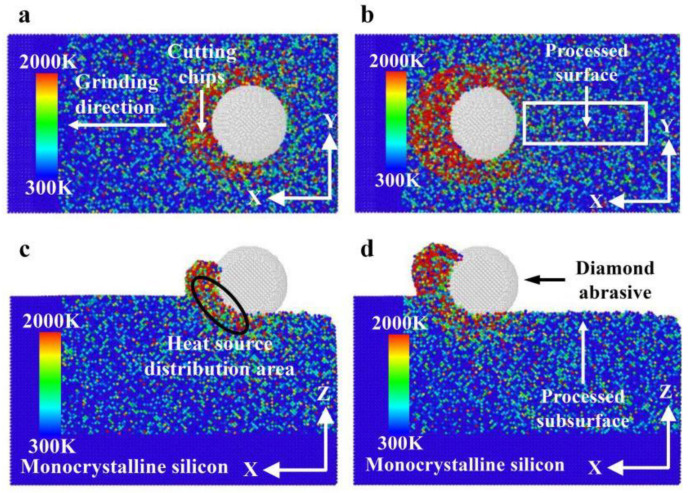
Heat distribution at different grinding distances (*d* = 2.0 nm, *v* = 200 m/s): (**a**) *x*-*y* surface with *l* = 8 nm; (**b**) *x*-*y* surface with *l* = 14 nm; (**c**) *x*-*z* surface with *l* = 8 nm; (**d**) *x*-*z* surface with *l* = 14 nm.

Figure 6 illustrates the heat distribution with different grinding distances during nano-grinding of monocrystalline silicon. According to Figure 6, with increasing grinding distance, the heat distribution in the grinding area mainly accumulates near the cutting chips and the abrasive grains. The heat distribution in the cutting chips is significantly higher than that near the abrasive grains, while the heat in front of and below the abrasive grains is higher than that behind them. The reason may be that the heat in the cutting chips includes the friction heat between the cutting chips and the abrasive grains, as well as the shear heat between an abrasive grain and monocrystalline silicon. Meanwhile, the heat near the abrasive grains mainly includes the heat generated in friction and in deformation between an abrasive grain and monocrystalline silicon. As the grinding progresses, the heat of the machining surface is gradually dissipated, so the heat basically acts on the contact area.

#### 3.1.2. Effect of Grinding Depth on Force–Thermal Behavior

**Figure 7:**
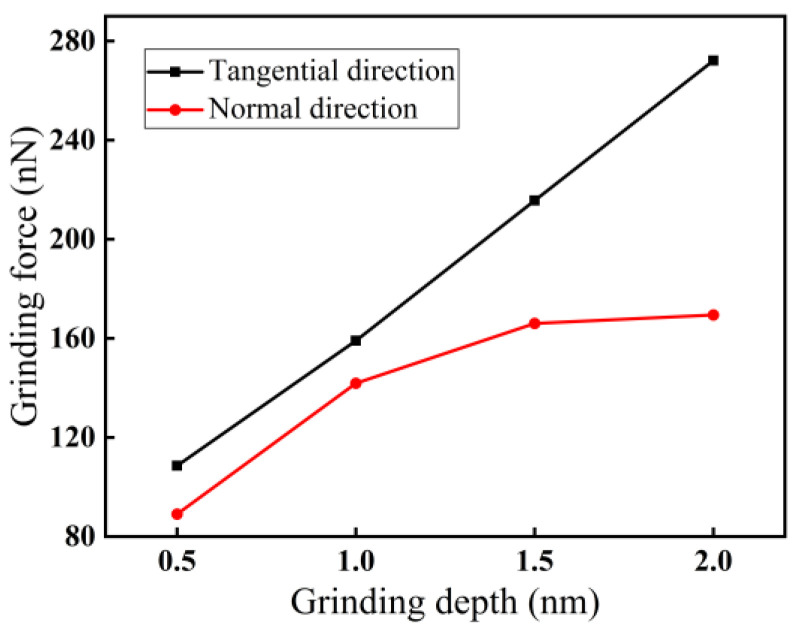
Variations in grinding force with grinding depth (*l* = 20 nm, *v* = 200 m/s).

The force–thermal behavior has a crucial effect on the formation of subsurface damage during nano-grinding of monocrystalline silicon. The force–thermal behavior and subsurface damage in nano-grinding of monocrystalline silicon can be changed by adjusting different machining parameters. For a grinding speed of 200 m/s, the effect of grinding depth on grinding force at a grinding distance of 20 nm is demonstrated in Figure 7. According to Figure 7, with increasing grinding depth, both the normal and tangential forces show an upward trend, and the force in the tangential direction is always larger than that in the normal direction. For a grinding depth of 2.0 nm, the difference between the two forces is the largest, about 103 nN. The reason may be that the contact arc length between an abrasive grain and monocrystalline silicon lengthens with increasing grinding depth, causing an increase in the extrusion strength of monocrystalline silicon and thus an increase in grinding force.

**Figure 8:**
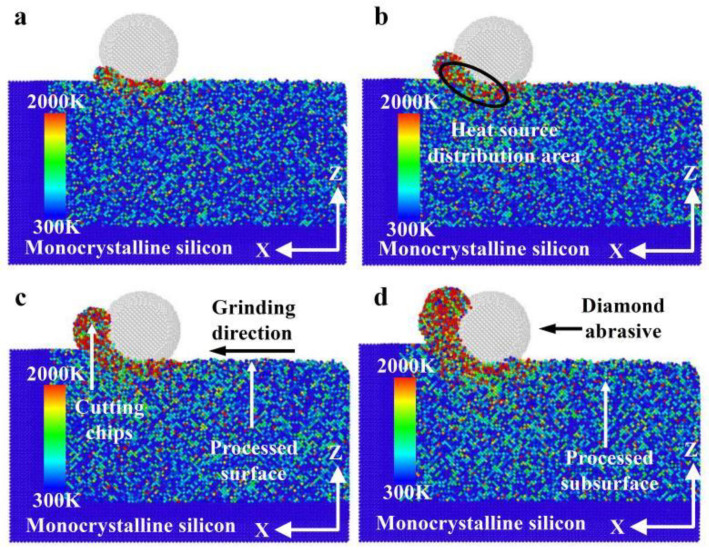
Heat distribution at different grinding depths (*l* = 20 nm, *v* = 200 m/s): (**a**) *d* = 0.5 nm; (**b**) *d* = 1.0 nm; (**c**) *d* = 1.5 nm; (**d**) *d* = 2.0 nm.

Figure 8 demonstrates the heat distribution in the grinding zone at different grinding depths during monocrystalline silicon nano-grinding. As demonstrated in Figure 8, with increasing grinding depth, the degree of thermal diffusion in the grinding area, as well as the heat below the abrasive grains and at the cutting chips, increases, and the number of cutting chips also increases accordingly. The reason may be that the contact area between an abrasive grain and monocrystalline silicon expands with increasing grinding depth, so the heat cannot be dissipated and is gathered in the contact area, resulting in an increase in heat in the grinding area. In addition, the amount of material removed per unit time increases with the increase in grinding depth, resulting in a corresponding increase in the amount of cutting chips.

**Figure 9:**
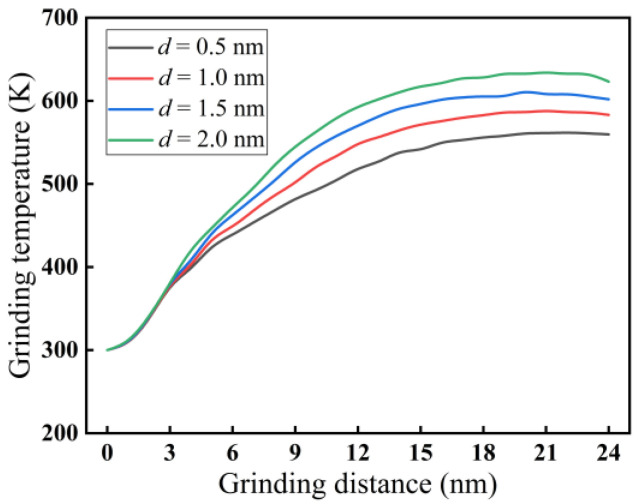
Variation curves of grinding temperature at different grinding depths (*v* = 200 m/s).

For a grinding speed of 200 m/s, the effect of grinding depth on grinding temperature is demonstrated in Figure 9. According to Figure 9, as the grinding depth increases within the range of 0.5 nm to 2.0 nm, the grinding temperature also rises. When the grinding distance reaches 24 nm, the grinding temperature rises from 560 K to 623 K for different grinding depths. The reason may be that the increased grinding depth leads to an increase in the contact arc length between the abrasive grains and monocrystalline silicon, causing a large amount of heat to enter the material. Therefore, the grinding temperature increases.

#### 3.1.3. Effect of Grinding Speed on Force–Thermal Behavior

**Figure 10:**
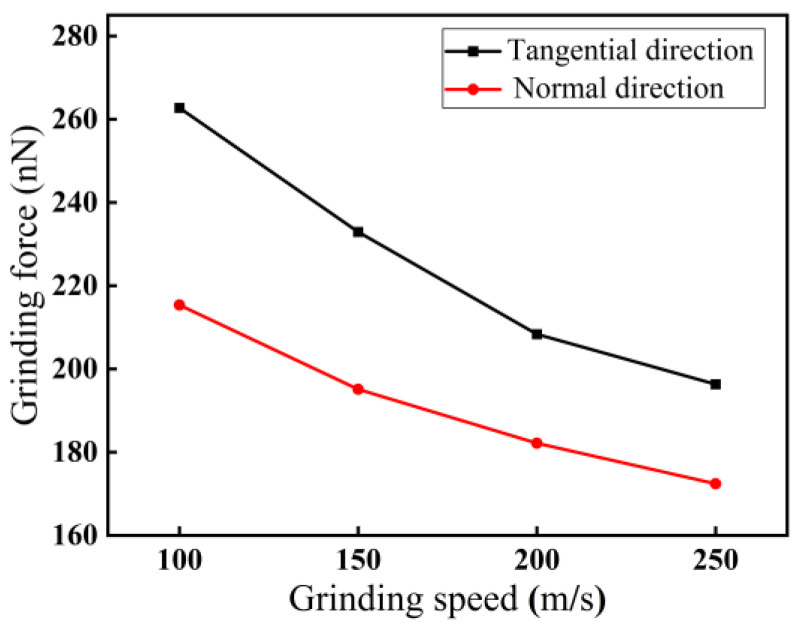
Variations in grinding force with grinding speed (*d* = 2.0 nm, *l* = 14 nm).

For a grinding depth of 2.0 nm, the impact of grinding speed on grinding force at a grinding distance of 14 nm is illustrated in Figure 10. As demonstrated in Figure 10, with increasing grinding speed from 100 m/s to 250 m/s, both the normal and tangential forces show a decreasing trend, while the force in the tangential direction remains larger than that in the normal direction. For a grinding speed of 250 m/s, the minimum difference is 24 nN. The reason may be that with increasing grinding speed, the contact area between an abrasive grain and monocrystalline silicon per unit time decreases, thereby reducing the actual removal thickness of the material in nano-grinding, resulting in a decreasing trend in grinding force.

**Figure 11:**
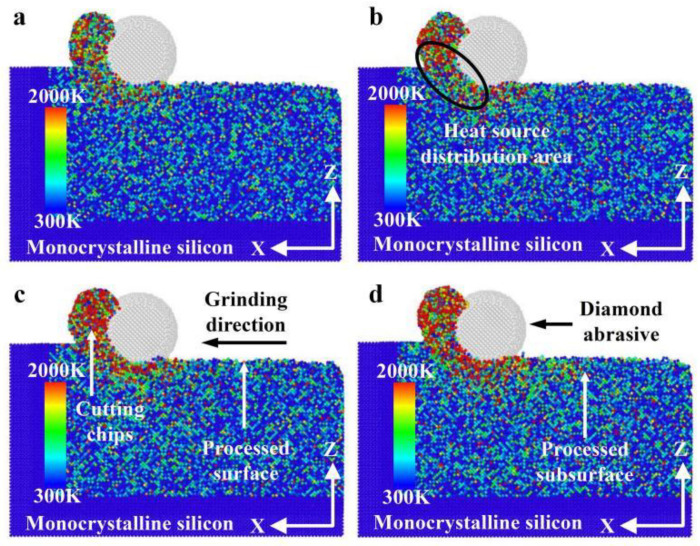
Heat distribution at different grinding speeds (*d* = 2.0 nm, *l* = 20 nm): (**a**) *v* = 100 m/s; (**b**) *v* = 150 m/s; (**c**) *v* = 200 m/s; (**d**) *v* = 250 m/s.

For a grinding depth of 2.0 nm and a grinding distance of 20 nm, the heat distribution at different grinding speeds is presented in Figure 11. As demonstrated in Figure 11, with increasing grinding speed, the heat distribution in the grinding area of monocrystalline silicon gradually expands, and the amount of cutting chips also increases. The reason may be that the rapid increase in grinding speed causes a significant portion of the heat generated in the contact area to be retained there before it begins to dissipate or dissipates completely. The heat cannot be dissipated in time with increasing grinding speed, resulting in the accumulation of most of the heat in the grinding area, and this accumulation effect expands the heat distribution.

**Figure 12:**
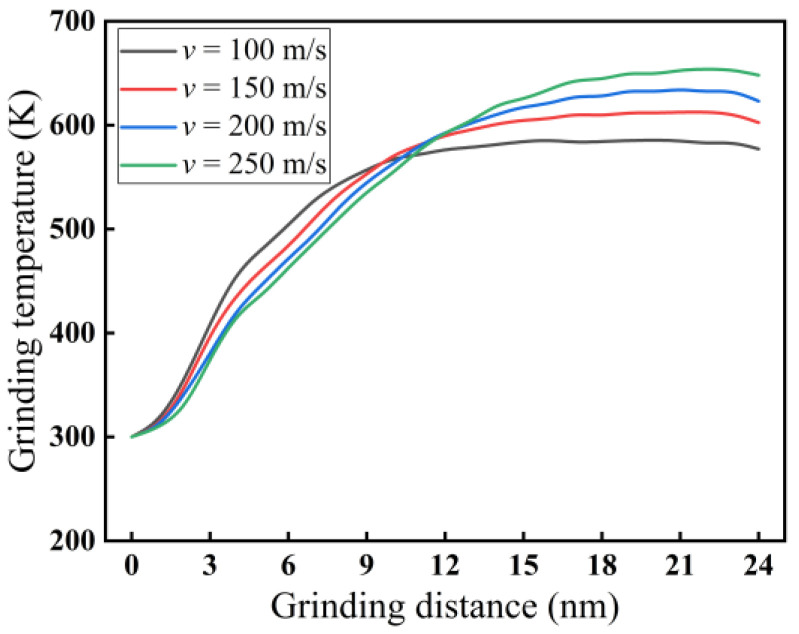
Variation curves of grinding temperature at different grinding speeds (*d* = 2.0 nm).

For a grinding depth of 2.0 nm, the impact of grinding speed on grinding temperature is demonstrated in Figure 12. As shown in Figure 12, the temperature increases throughout the grinding distance range (0–24 nm). Notably, at higher grinding speeds, lower temperatures were achieved (0–9 nm). The situation is different for grinding distances of 12 to 24 nm, where higher grinding speeds result in higher temperatures. The intersections of grinding temperatures occur within a grinding distance of 9 to 12 nm. When the grinding distance is 24 nm, the grinding temperature rises from 577 K to 648 K as the grinding speed increases from 100 to 250 m/s. The reason may be that in an incipient stage of nano-grinding processes, less grinding heat is generated, and the grinding temperature is relatively low, so the heat dissipation is fast. With the increase in grinding speed, the grinding heat is dissipated by the abrasive grains, leading to a reduction in grinding temperature and a tendency towards stability. However, during the stable grinding period beyond 12 nm, the grinding temperature is already relatively high, and the heat dissipation is slow. Due to the increase in grinding speed, the abrasive grains exert a violent and rapid squeezing effect on monocrystalline silicon, breaking a large number of atomic bonds in monocrystalline silicon. Thus, more heat is generated and there is not enough time for dissipation, resulting in an increase in grinding temperature.

### 3.2. Analysis of Subsurface Damage for Monocrystalline Silicon in Nano-Grinding

#### 3.2.1. Formation Mechanism for Subsurface Damage

**Figure 13:**
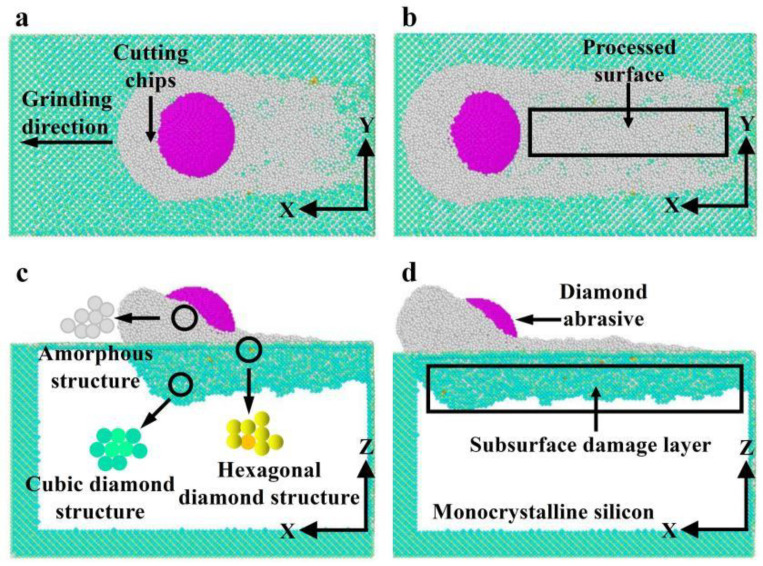
Subsurface damage at different grinding distances (*d* = 2.0 nm, *v* = 200 m/s): (**a**) *x*-*y* surface with *l* = 16 nm; (**b**) *x*-*y* surface with *l* = 23 nm; (**c**) *x*-*z* surface with *l* = 16 nm; (**d**) *x*-*z* surface with *l* = 23 nm.

In nano-grinding processes, under the action of force–thermal behavior and friction, the workpiece undergoes deformation, leading to subsurface damage. Figure 13 shows the subsurface damage in monocrystalline silicon in nano-grinding at different grinding distances. The atomic structure in Figure 13 is obtained by coloring and analyzing a diamond structure in the visualization software. In order to facilitate analysis, the monocrystalline silicon with a perfect structure after grinding is hidden. As demonstrated in Figure 13, the monocrystalline silicon generates atoms with three different structures under the action of force in the nano-grinding: cubic diamond neighboring structure, hexagonal diamond structure, and amorphous structure. Among them, the amorphous structures mainly appear on the cutting chips and processed surfaces. The hexagonal diamond structures are mainly distributed in or near amorphous regions, while the cubic diamond neighboring structures are mainly distributed on the ground subsurface of the workpiece, at smaller scales compared to the amorphous structure atoms. This indicates that the formation mechanisms of subsurface damage in monocrystalline silicon in nano-grinding are mainly phase transformation and amorphization.

In addition, amorphous structures appear above the cubic diamond neighboring structures, while hexagonal diamond structures are rare there. This is because the temperature near the processed surface is high, and under the action of high temperature, the monocrystalline silicon can easily form amorphous structures [1]. The formation of hexagonal diamond structures, a phase-transition structure, requires specific critical loads. When the stress value exceeds the critical stress value for phase transition in monocrystalline silicon, atoms of the phase-transition structure are generated [7]. The high heat distribution also provides energy support for the generation of phase transitions. For amorphous structures, which are mainly generated in chips and processed surfaces, the atomic displacement of the chips and processed surfaces is greatest during the grinding process, which will result in larger-scale plastic deformation.

#### 3.2.2. Effect of Grinding Depth on Subsurface Damage

**Figure 14:**
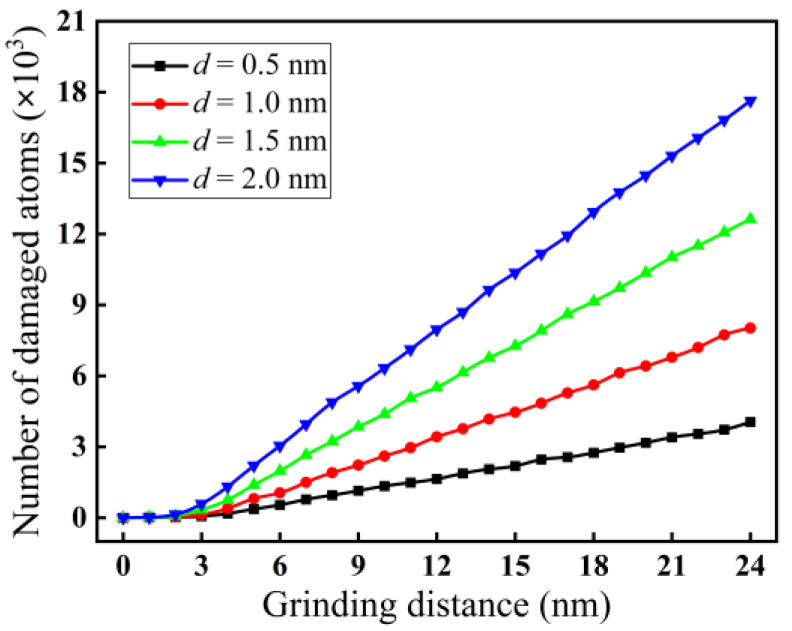
Variation curves of the quantity of damaged atoms at different grinding depths (*v* = 200 m/s).

In nano-grinding of monocrystalline silicon, the change in grinding parameters will affect the subsurface damage. For a grinding speed of 200 m/s, the variation curves of the quantity of damaged atoms with grinding depth are shown in Figure 14. As demonstrated in Figure 14, when the grinding depth is in the range of 0.5 to 2.0 nm, the quantity of damaged atoms increases with grinding depth. At a grinding distance of 24 nm, the number of damaged atoms increases from 4.049 × 10^3^ to 17.637 × 10^3^ with increasing grinding depth from 0.5 to 2.0 nm. The reason may be that with increasing grinding depth, the abrasive particle cuts deeper into the interior of the material and removes the material through shear and squeezing. Thus, the contact area between the abrasive particle and the workpiece increases, and the grinding force also increases, causing a rise in the internal temperature of the deformation zone. As a result, the motion of atoms becomes more intense, which reduces the energy required for the atomic phase transition, causing an increase in the quantity of damaged atoms.

**Figure 15:**
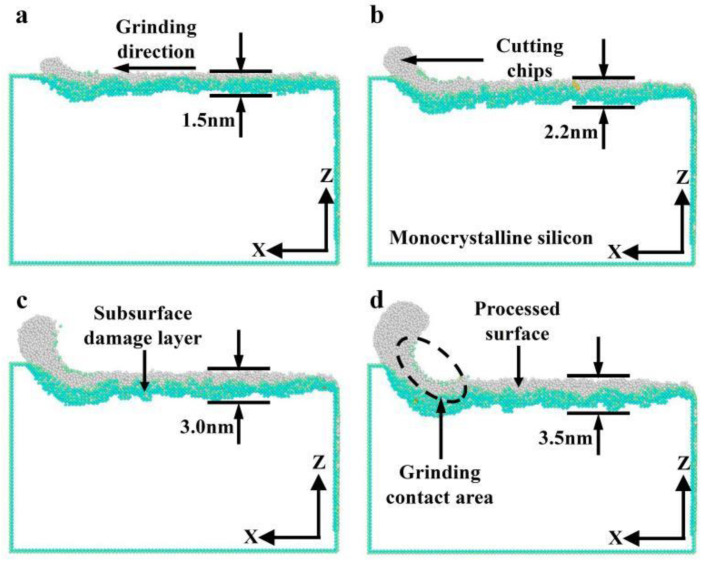
Subsurface damage at different grinding depths (*v* = 200 m/s): (**a**) *d* = 0.5 nm; (**b**) *d* = 1.0 nm; (**c**) *d* = 1.5 nm; (**d**) *d* = 2.0 nm.

Figure 15 illustrates the distribution of subsurface damage in monocrystalline silicon at different grinding depths in nano-grinding. According to Figure 15, with increasing grinding depth from 0.5 to 2.0 nm, the thickness of the subsurface damaged layer of monocrystalline silicon increases from 1.5 to 3.5 nm. Its change trend coincides with the change in the number of damaged atoms in the subsurface. The reason may be that both the force and heat grow with increasing grinding depth, and a large grinding force accelerates the formation rate of subsurface damage in the workpiece.

#### 3.2.3. Effect of Grinding Speed on SUBSURFACE Damage

**Figure 16:**
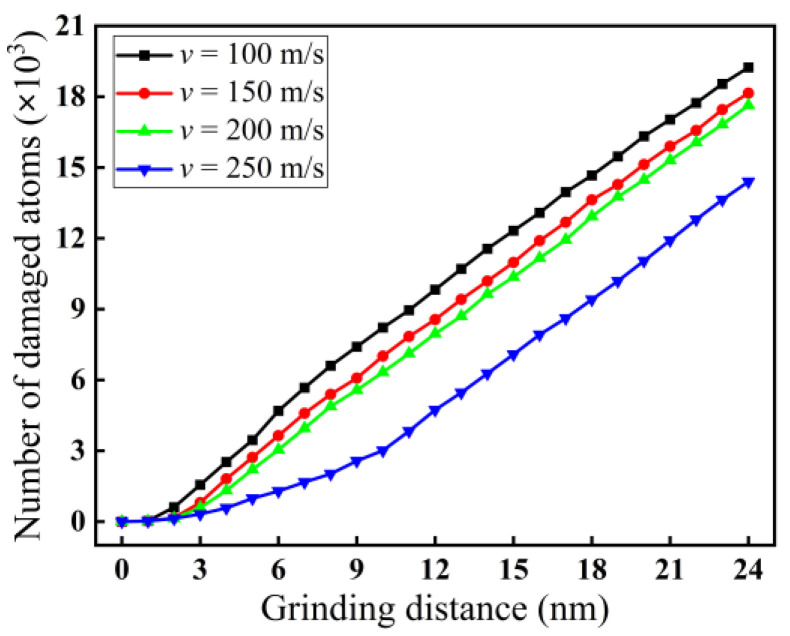
Variation curves of the quantity of damaged atoms at different grinding speeds (*d* = 2.0 nm).

For a grinding depth of 2.0 nm, the variation curves of the quantity of damaged atoms with grinding speed are shown in Figure 16. As demonstrated in Figure 16, the quantity of damaged atoms decreases with the increase in grinding speed from 100 to 250 m/s. When the grinding distance is 24 nm, the number of damaged atoms decreases from 19.243 × 10^3^ to 14.401 × 10^3^. The reason may be that with increasing grinding speed, the contact time between the abrasive particle and the surface of the workpiece being processed is shortened, reducing heat accumulation and decreasing the possibility of thermal damage to the monocrystalline silicon. On the other hand, although the grinding temperature also rises with increasing grinding speed, the grinding force decreases, resulting in fewer atoms being damaged. In addition, for monocrystalline silicon, a typical hard, brittle, and difficult-to-machine material, a certain rise in grinding temperature enhances the toughness of the material, which is beneficial for improving the plastic removal ratio of silicon. This reduces the quantity of damaged atoms, resulting in a smaller subsurface damage layer.

**Figure 17:**
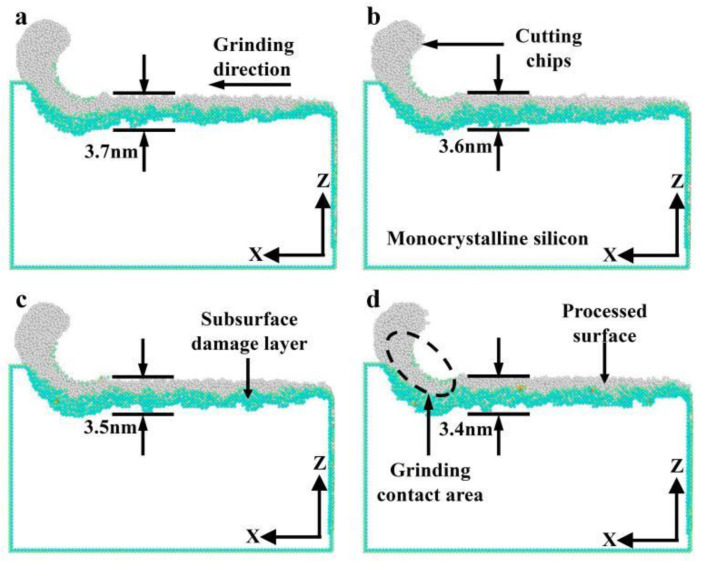
Subsurface damage at different grinding speeds (*d* = 2.0 nm): (**a**) *v* = 100 m/s; (**b**) *v* = 150 m/s; (**c**) *v* = 200 m/s; (**d**) *v* = 250 m/s.

Figure 17 presents the distribution of subsurface damage in monocrystalline silicon at different grinding speeds in nano-grinding. As illustrated in Figure 17, with increasing grinding speed from 100 to 200 m/s, the thickness of the subsurface damaged layer of monocrystalline silicon increases from 3.7 to 3.4 nm. Its change trend is also consistent with the change in the number of damaged atoms in the subsurface. The reason may be that as grinding speed increases, the shear stress gradually decreases and the grinding heat increases. However, a certain degree of high temperature strengthens the toughness of the material, causing a relative improvement in the subsurface quality of monocrystalline silicon [34,35].

### 3.3. Analysis of Potential Energy

In order to better understand the behavior and characteristics of materials during grinding, especially the impact on grinding efficiency and quality, the analysis of potential energy is particularly important. Potential energy can accurately reflect the change in internal energy caused by the change in atomic position or arrangement in the workpiece. By analyzing the changes in potential energy, the material removal mechanism of monocrystalline silicon in nano-grinding can be identified. Potential energy analysis also helps reveal the influence of key parameters such as grinding speed and grinding depth on processed surface quality, thereby optimizing grinding parameters to improve machining performance. In addition, the analysis of potential energy helps predict stability during the grinding process, reducing vibration and other instabilities, thereby ensuring the quality of the machined surface. Therefore, analysis of potential energy not only helps optimize process conditions but also improves the overall processing efficiency and surface quality of monocrystalline silicon.

**Figure 18:**
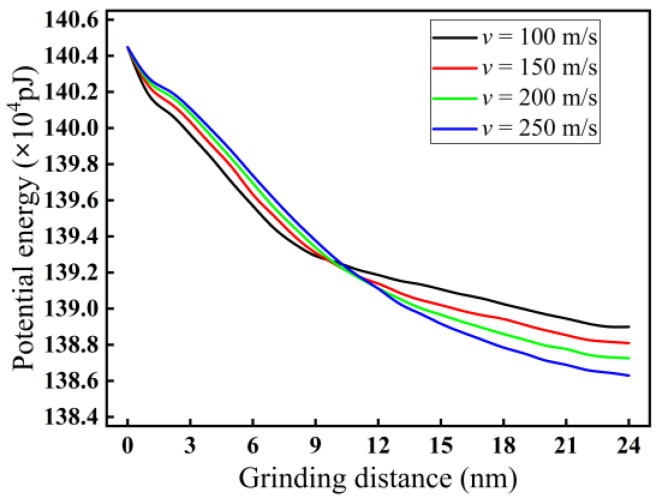
Variation curves of potential energy at different grinding speeds (*d* = 2.0 nm).

Figure 18 displays the impact of grinding speed on potential energy in nano-grinding of monocrystalline silicon. As demonstrated in Figure 18, as the grinding speed increases from 100 to 250 m/s, the potential energy between silicon atoms first increases and then decreases. Because the crossover of potential energy between different grinding speeds occurs at different grinding distances, the crossing points occur at a grinding distance of approximately 10 nm instead of a fixed distance. This is similar to the change in temperature. The reason may be that in the initial stage of nano-grinding, with increasing grinding speed, the contact frequency and squeezing effect between an abrasive grain and monocrystalline silicon increase, leading to more atomic bonds being broken. The enhanced interaction between silicon atoms results in an increase in potential energy. However, as the nano-grinding progresses, a more stable surface layer gradually forms on the surface of monocrystalline silicon, and the interaction between silicon atoms correspondingly decreases. With increasing grinding speed, potential energy undergoes a crossover. Therefore, in a stable nano-grinding state after a distance of 12 nm, increasing the grinding speed decreases the potential energy between silicon atoms.

**Figure 19:**
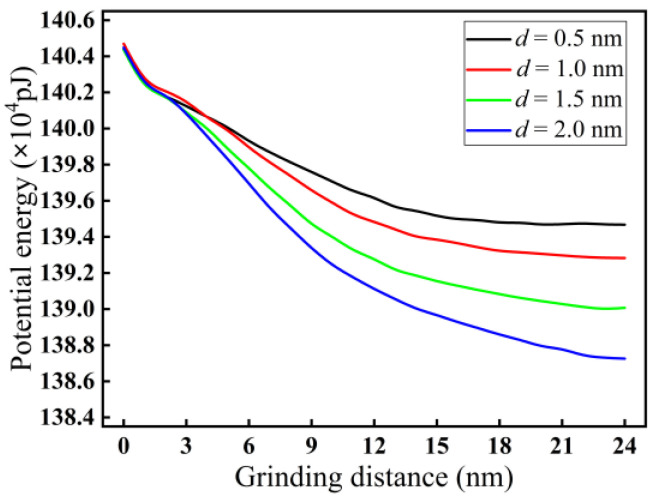
Variation curves of potential energy at different grinding depths (*v* = 200 m/s).

Figure 19 presents the variations in potential energy with grinding depth in nano-grinding of monocrystalline silicon. As illustrated in Figure 19, the increase in grinding distance gradually reduces and stabilizes the intermolecular potential energy. At a stable stage of grinding, with increasing grinding depth from 0.5 to 2.0 nm, the potential energy between silicon atoms gradually decreases. The reason may be that with increasing grinding depth, the removal rate of silicon atoms and grinding temperature increase, which leads to changes in surface structure and enhanced thermal vibration of silicon atoms, resulting in a decrease in potential energy.

In addition, Ref. [36] adopted an MD model to investigate the material removal mechanism of silicon carbide in nanocutting by setting the cutting speed at [100, 400] m/s, the cutting depth at [0.5, 5.0] nm, the cutting distance at [0, 24] nm, and the time step at 1 fs. Then, nanocutting experiments were carried out using a piezoelectric ceramic nano mobile station with a vacuum environment in a scanning electron microscope. The results show that the simulation model is highly consistent with the experimental results, which effectively confirms the reliability of the simulation model. Considering that the parameter settings of the simulation process in Ref. [36] are similar to those in this study, the experimental results provide strong support for the reliability of the simulation results of this study.

## 4. Conclusions

The subsurface formation mechanism in monocrystalline silicon under nano-grinding was investigated using MD simulations. The impacts of grinding parameters on the force–thermal behavior and the mechanism of subsurface damage, as well as the change in potential energy during nano-grinding, were analyzed. This research provides a theoretical foundation for the suppression of subsurface damage in monocrystalline silicon. The conclusions are as follows:

(1) Among the three grinding forces—tangential, lateral, and normal—the lateral grinding force fluctuates around zero and can be ignored. The grinding force in the tangential direction is slightly higher than that in the normal direction and plays a crucial role in material removal. Grinding force, shear stress, and friction coefficient all increase with increasing grinding depth and decreasing grinding speed.
(2) The heat in the grinding area mainly accumulates near the cutting chips and abrasive particles, and the heat in the chips is higher than the heat distribution near the abrasive grains. The heat in front of and below the abrasive grains is higher than that behind them. The grinding temperature rises with increasing grinding depth. At the incipient stage of nano-grinding of monocrystalline silicon, the higher the grinding speed, the lower the grinding temperature. However, in the stable grinding stage beyond a distance of 12 nm, the grinding temperature shows an increasing trend with increasing grinding speed. With increasing grinding speed, the potential energy between silicon atoms shows an opposite trend compared to the grinding temperature.
(3) Both phase transformation and amorphization are the main formation mechanisms of subsurface damage in nano-grinding of monocrystalline silicon. The quantity of damaged atoms and the thickness of the subsurface damage layer increase with increasing grinding depth and decreasing grinding speed. Therefore, in nano-grinding processes, reducing the grinding depth (e.g., a small grinding depth of 0.5 nm) and increasing the grinding speed (e.g., a high grinding speed of 250 m/s) should be prioritized to effectively suppress subsurface damage in monocrystalline silicon.

## Figures and Tables

**Table 1:** Specific parameters for simulation.

Simulation Parameter	Value
Workpiece size (nm × nm × nm)	26 × 14 × 15
Total number of atoms, *N*	296,936
Lattice constant of workpiece (nm)	0.543
Lattice constant of diamond (nm)	0.357
Diamond abrasive radius, *r* (nm)	3
Grinding depth, *d* (nm)	0.5, 1.0, 1.5, 2.0
Grinding speed, *v* (m/s)	100, 150, 200, 250
Grinding distance, *l* (nm)	0–24
Grinding direction	[−100] (100)
Time step, *t* (fs)	1

## Data Availability

The data presented in this study are available in the article.
